# Space radiation health risks to hematopoietic, neural, and gastrointestinal systems in astronauts

**DOI:** 10.3389/fpubh.2026.1926972

**Published:** 2026-09-07

**Authors:** Longzhen Zhang, Yishu Yin, Runze Li, Ge Wang, Liang Li, Yingying Yu, Weihong Lu

**Affiliations:** 1 School of Medicine and Health, Harbin Institute of Technology, Harbin, China; 2 Zhengzhou Advanced Research Institute, Harbin Institute of Technology, Zhengzhou, China; 3 Institute of Space Environment and Material Science, Harbin Institute of Technology, Harbin, China

**Keywords:** gastrointestinal system, hematopoietic system, neural system, space radiation, spaceflight

## Abstract

The rapid progress in global deep space exploration presents astronauts with a complex space radiation environment that substantially exceeds that of near-Earth orbit. Space radiation primarily consists of galactic cosmic rays (GCRs) and solar particle events (SPEs), which exhibit strong penetrative capability and considerable biological damaging potential. These factors may induce persistent oxidative stress, mitochondrial dysfunction, DNA double-strand breaks, and repair pathway challenges. This review examines the impacts of space radiation on the hematopoietic, neural, and gastrointestinal systems. Human spaceflight observations indicate that exposure to space radiation may be associated with bone marrow suppression, reduced lymphocyte counts, diminished thymic output, and immune dysfunction, which may heighten susceptibility to infection and inflammation. Preclinical animal model research indicates that even low doses of space-relevant radiation may induce cognitive decline, memory deficits, anxiety-like behaviors, and neurodegenerative alterations, though these findings remain to be validated in human astronauts. Proposed mechanisms include impaired hippocampal synaptic plasticity, neuroinflammatory activation, and mitochondrial energy metabolism imbalance. Radiation has been shown to compromise the intestinal epithelial barrier, induce intestinal stem cell senescence, and disturb gut microbiome equilibrium in preclinical models. These gastrointestinal alterations may potentially exacerbate neurocognitive dysfunction and promote chronic inflammation via the gut-brain axis, based on mechanistic evidence from animal studies. Currently, there remains an insufficient systematic understanding of the cumulative effects, sex differences, and individual susceptibility associated with long-term low-dose composite radiation exposure. Integrating aerospace medicine is imperative to address these knowledge gaps and safeguard astronaut health during future deep-space missions.

## 1 Introduction

Deep space exploration is a long-term goal pursued globally by space agencies. Manned lunar landing missions led by the United States (Artemis program) and China are scheduled for implementation by 2028 and 2030, respectively, with crewed Mars missions targeted for the mid-21st century. As missions extend beyond low Earth orbit into deep space, astronauts will face substantially elevated and more complex space radiation exposures, which represent a critical biological barrier to long-duration human deep space travel. Astronauts encounter an increased threat from space radiation upon exiting Earth's magnetic field and entering space. The natural background radiation dose equivalent rate received by the human body on the Earth's surface, shielded by the atmosphere and the Earth's magnetic field, is roughly 2.4 mSv/year, which poses a negligible acute and chronic health risk (1). Astronauts aboard the space station in low Earth orbit are not entirely shielded by the Earth's magnetic field, receiving an equivalent dose rate of up to 0.648 mSv/d (236.52 mSv/year equivalent dose), roughly 100 times greater than the equivalent dose on the Earth's surface. The radiation environment consists mainly of low-LET protons with a minor HZE contribution. During deep space travel, including lunar landings, astronauts are exposed to an equivalent dose rate of 57.1 ± 10.6μSv/h (500 mSv/year equivalent dose), which is 200 times greater than the dose received on Earth's surface. Future missions to Mars will lead to heightened radiation exposure, surpassing 350 mSv annually. Additionally, it should be noted that the above estimates reflect chronic GCR exposure only; unpredictable solar particle events can deliver acute high-dose exposure over a brief duration—for example, a major SPE event may deliver over 1 Sv of whole-body dose within several hours during extravehicular activity (2–4). Geant4-DNA mechanistic Monte Carlo simulations have evaluated multilayer shielding against extreme SPEs. The shelter reduced DNA damage by 36.7% and 57.9%, and lethal damage by 64.3% and 88.2%, respectively, while enhancing cell survival (5). In the future, humanity will persist in its exploration of Mars and galaxies beyond the solar system, leading astronauts to confront increasingly complex radiation hazards. While chronic galactic cosmic ray exposure delivers relatively low daily dose rates, unshielded exposure to intense SPEs can deliver life-threatening acute doses, particularly during extravehicular activities where spacesuit shielding provides limited protection. A radiation absorbed dose of 2 Sv indicates the onset of acute radiation syndrome, with a 35% mortality rate within 30 days. Without timely comprehensive clinical supportive intervention, uniform whole-body radiation at approximately 10 Sv equivalent dose usually triggers fatal multi-organ acute radiation syndrome, with nearly universal mortality within 7 days (6).

**Figure 1:**
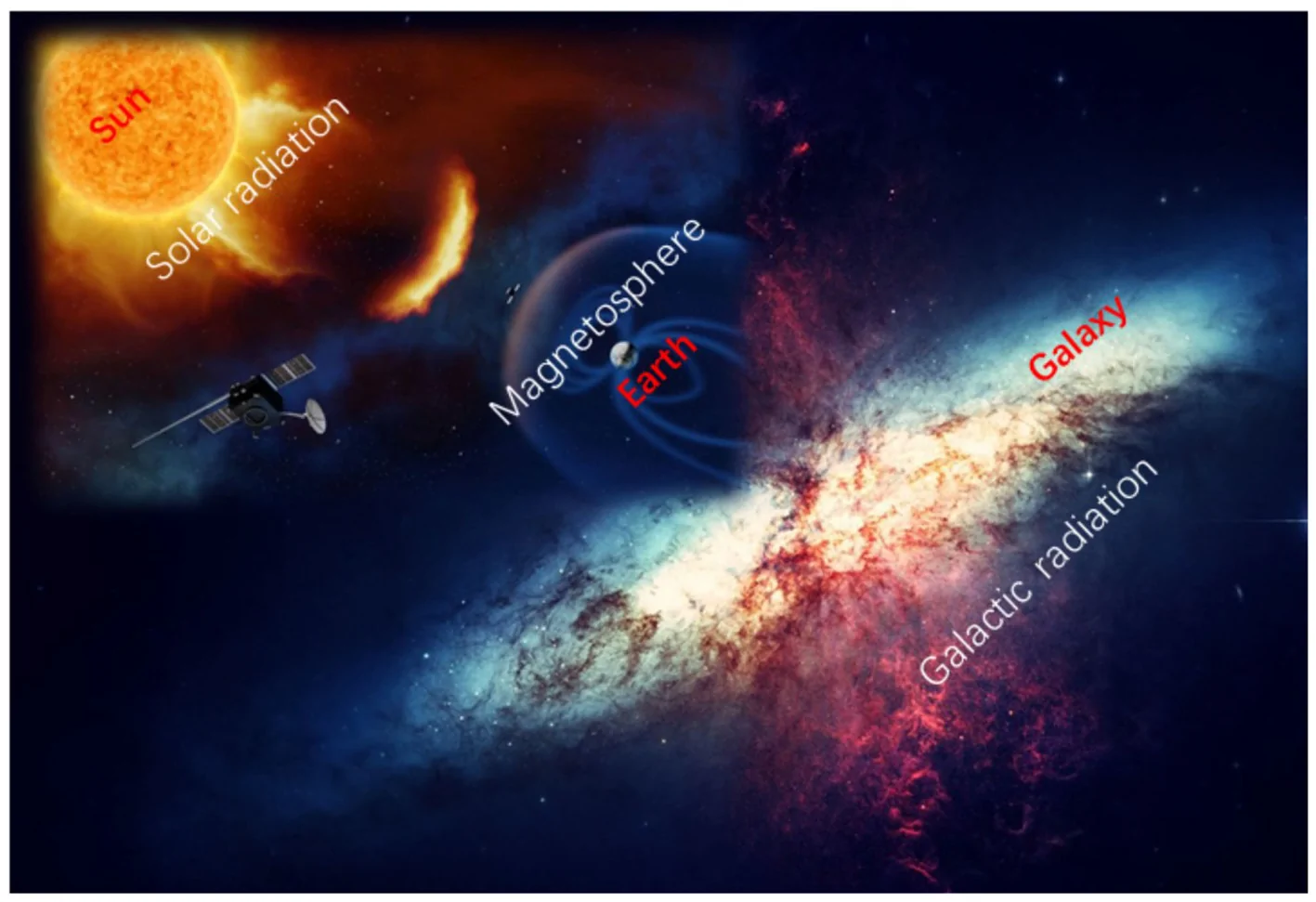
The principal types of space radiation.

Space radiation primarily comprises high-energy protons from SPEs, heavy ions present in GCRs, and secondary particles created through interactions with spacecraft shielding. GCRs constitute the primary source of radiation and the principal determinant of the biological impacts of space radiation (Figure 1) (7). The space radiation environment comprises a range of charged particles: electrons and positrons (2%), protons (85%), helium nuclei (12%), and high-energy protons and high charge (Z) and energy (E) nuclei (HZE) (1%) (1, 7, 8). The charged particles exhibit a broad spectrum of energy, spanning several orders of magnitude. This includes protons and electrons with an average energy of around 10 MeV, alongside high-energy heavy ions and protons with energies in the GeV range.

Linear energy transfer (LET) refers to the energy absorbed by charged particles for each unit of distance traveled within a material (9). Space radiation consists primarily of protons and helium nuclei, which dominate the fluence; HZE particles, although accounting for only a small fraction of the particle flux, exert a disproportionately large biological impact (Table 1) (10). Compared with low-LET radiation (e.g., γ-rays and X-rays)—which primarily inflicts diffuse, indirect DNA damage via water radiolysis and reactive oxygen species, yielding discrete and readily repairable lesions—high-LET radiation exhibits stronger penetrability and deposits energy far more densely along particle tracks, inflicting substantially more severe cellular damage. This dense ionization generates nanoscale clustered DNA lesions, including double-strand breaks (DSBs), single-strand breaks, abasic sites, and oxidized base adducts—a spectrum fundamentally distinct from the sparse, isolated damage characteristic of low-LET exposure. These complex clusters profoundly impair the fidelity of both non-homologous end joining (NHEJ) and Homologous recombination (HR) repair pathways: faulty end ligation via NHEJ gives rise to translocations and micronuclei, whereas oxidative base lesions block precise HR template matching, leaving persistent unrepaired DNA damage detectable months after exposure (11–13). Importantly, micronuclei arise from misrepaired acentric chromosome fragments that fail to integrate into mitotic spindles; these micronuclei entrap damaged DNA and perpetuate chronic oxidative signaling, thereby sustaining a self-amplifying cycle of genomic instability. Concurrently, track ionization triggers lasting mitochondrial electron leakage and sustained overproduction of ROS/RNS, establishing an additional oxidative feedback loop that continuously generates secondary DNA damage—a cascade barely observed under low-LET radiation (14). Collectively, unresolved complex DSBs, coupled with chronic oxidative stress, propagate delayed genomic instability across cell progeny, markedly raising long-time risks of somatic mutation and malignant transformation, a long-term heritable damage signature that remains negligible after standard low-LET radiation exposure (15, 16). These impairments could present in various systems of the human body, including the hematopoietic, neural, and gastrointestinal systems (7, 17, 18). While space radiation-induced biological damage is well-documented, systematic analyses of multi-organ cascade effects (hematopoietic, neural, and gastrointestinal) under long-term low-dose combined exposure remain limited. Therefore, elucidating these sequential damage patterns is essential for addressing current knowledge gaps and informing radiation protection strategies for deep-space astronauts.

**Table 1:** Comparative analysis of low-LET and high-LET radiation.

LET	Particle Class	Type	LET (keV/μm)	ICRP 103 *w_*R*_*	NCRP 132 Q(LET)
Low	γ-Ray	^60^Co γ-Rays	0.1–0.4	1	1.0
	X-Ray	250 Kv X-Rays	0.5–3.0	1	1.0
	β-particle	0.5 MeV β^−^	0.2–4.0	1	1.0
	Proton	10 MeV Protons	2.0–8.0	2	~1.8
High	Neutron	14 MeV Neutrons	10.0–30.0	7–10^1^	7.0^2^
	Proton	70 MeV Protons	5.0–50.0	2^3^	4.2^2^
	α-Ray	2.5 MeV α	80.0–200.0	20^3^	23.2^2^
	Heavy ion	2 GeV Fe ions	200.0–2,000.0	20^3^	9.5^2^

Data sources: ICRP 103, NCRP 132.

^1^In ICRP 103, the radiation weighting factor w_R_ for neutrons is not a fixed value but a continuous function of neutron energy, peaking at about 20 at approximately 1 MeV and decreasing at both low and high energies. The w_R_ value for 14 MeV neutrons in the table lies roughly in the range of 7–10, depending on the specific energy spectral distribution.

^2^In NCRP 132, Q(LET) is a continuous function of LET. The Q values given in the table are spectrum-averaged approximations along the particle track over its LET range; in practical applications, integral calculations over the particle energy spectrum and LET distribution are required. For example, the LET of a 2 GeV Fe ion varies from ~200 keV/μm to >2,000 keV/μm along the track, corresponding to Q values decreasing from ~21.2 down to ~6.7; the tabulated value of 9.5 is a representative average for this track.

^3^ICRP 103 assigns a uniform w_R_ = 2 to protons (all energies) and a uniform w_R_ = 20 to alpha particles and heavy ions (all energies and charge numbers). These values are discrete, step-wise recommended values and do not vary continuously with LET or energy.

## 2 Impact of space radiation on the hematopoietic system

### 2.1 The hematopoietic system as a primary radiation-vulnerable organ

**Figure 2:**
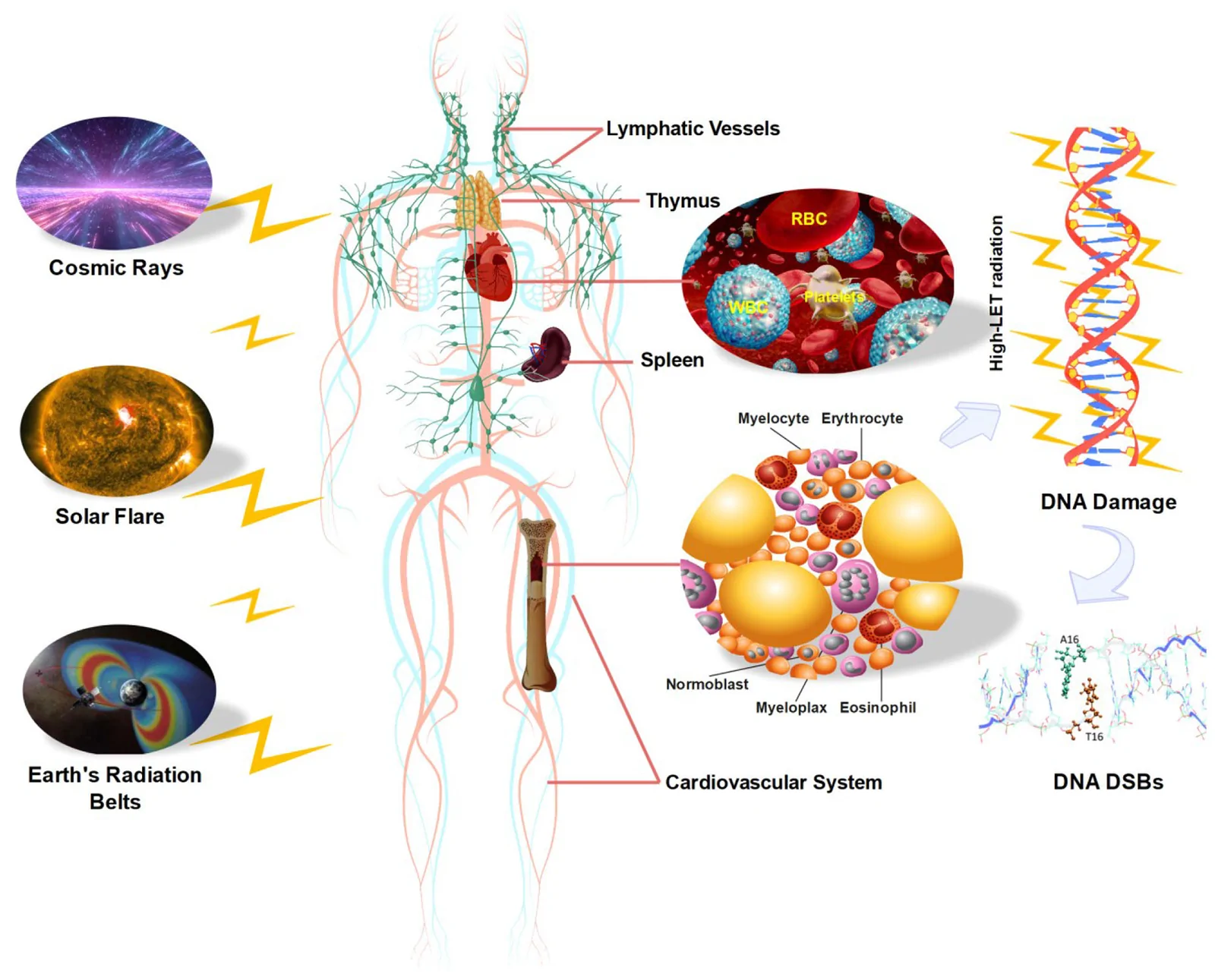
Impact of space radiation on the hematopoietic system.

The hematopoietic system includes both blood and the hematopoietic system, comprising the bone marrow, spleen, thymus, and lymphatic tissue. It is one of the most vulnerable systems in the human body to space radiation (Figure 2) (19, 20). Its extreme vulnerability to ionizing radiation is fundamentally attributed to the high continuous cellular turnover characteristic of hematopoietic lineages. Unlike quiescent or slowly dividing somatic cells, blood cells possess relatively short lifespans and require persistent proliferation and differentiation of pluripotent hematopoietic stem cells (HSCs) to sustain peripheral circulation homeostasis (21). Specifically, human red blood cells survive approximately 120 days, platelets persist for 7–10 days, and most circulating white blood cells only remain functional for several hours to days. This rapid replacement cycle forces sustained DNA replication and cell division in HSCs and progenitor cells, rendering hematopoietic populations inherently susceptible to genotoxic stress induced by high-linear energy transfer (high-LET) space radiation.

Space radiation exerts rapid, severe, and dose-dependent detrimental effects on hematopoietic homeostasis. High-LET radiation directly induces complex DNA lesions in proliferative HSCs and downstream progenitors, disrupting ordered differentiation programs, impairing mature blood cell production, and triggering widespread cellular dysfunction or apoptosis (22, 23). Compared with terrestrial low-LET radiation, deep-space high-LET particles produce clustered DNA damage that is difficult to repair, further exacerbating persistent hematopoietic suppression and predisposing the organism to subsequent systemic immune impairment.

### 2.2 Hematopoietic stem cell depletion and bone marrow niche disruption induced by space radiation

Beyond acute peripheral blood cell loss, space radiation elicits persistent hematopoietic dysfunction primarily through HSC genotoxic damage, proliferative arrest, senescence, and apoptosis, accompanied by comprehensive destruction of the bone marrow microenvironment. High-LET radiation-induced complex DNA damage severely compromises HSC genomic stability, leading to cell cycle arrest, senescence activation, and progressive depletion of functional HSC pools, which severely impairs the self-renewal and multi-lineage differentiation capacity required for long-term hematopoietic reconstruction.

Compounding HSC injury, space radiation triggers profound structural and functional remodeling of the bone marrow niche. Bone marrow adipose tissue, an active endocrine and immunomodulatory component critical for regulating HSC fate, undergoes significant remodeling following spaceflight and radiation exposure. Long-duration ISS mission analyses suggested significant reductions in bone marrow adipose tissue among astronauts' post-flight, with lumbar vertebral marrow adipose levels positively correlated with peripheral red blood cell density, suggesting that niche adipocyte remodeling is closely associated with hematopoietic functional alterations (24). In addition, radiation induces endothelial injury within the perivascular niche, which serves as the core microenvironment supporting HSC residence and renewal. Vascular endothelial dysfunction further disrupts niche cytokine signaling networks, abrogating the balanced paracrine regulation essential for HSC self-renewal, quiescence maintenance, and lineage differentiation.

Consistent with human observational evidence, ground simulation studies have validated radiation-mediated bone marrow suppression. Whole-body carbon ion radiation exposure in mice significantly reduced peripheral blood erythrocytes, lymphocytes, granulocytes, and monocytes, accompanied by drastically altered bone marrow transcriptomic profiles that directly drive hematopoietic failure (25). Collectively, space radiation triggers dual damage to both HSCs and their supporting niche microenvironment, resulting in hematopoietic homeostasis disruption.

### 2.3 Multi-source evidence of hematopoietic radiation injury

Human spaceflight data offer valuable observational evidence, albeit confounded by concurrent stressors, which complicates direct attribution of hematopoietic alterations solely to radiation. The landmark NASA Twins Study revealed that one-year spaceflight exposure significantly elevated systemic inflammatory chemokines and inflammatory markers (IL1A, IL1B, IL2, PGF2α), indicative of persistent hematopoietic and immune activation stress (26). Peripheral blood analyses of long-duration astronauts further observed elevated circulating CD4 and CD8 lymphocyte subsets and increased leukocyte telomere length during space missions, indicating that space environmental exposure is associated with remodeling of human peripheral blood immune cell profiles. Recent transcriptomic and epigenetic analyses of astronaut peripheral blood samples further identified significant in-flight monocyte depletion, a hallmark of suppressed bone marrow hematopoietic output and subclinical immune deficiency (27). These human in-flight observations collectively verify that space environment exposure induces measurable hematopoietic and immune alterations, even under LEO partially shielded conditions.

Given the limitations of human space sampling, ground-based simulated GCR and high-LET radiation animal models have become the primary approach to elucidate dose-dependent and sex-specific hematopoietic injury mechanisms. Simulated galactic cosmic radiation exposure (50 cGy) induced significant increases in monocyte (CD11bCD45) and natural killer cell (NK1.1CD45) populations in male mice, while B-cell populations remained relatively stable; notably, f female mice exhibited no significant peripheral blood alterations under identical radiation conditions, suggesting sex-dependent hematopoietic radiosensitivity under the specific experimental conditions (28). High-dose gamma radiation (7–11 Gy Cs) in C57BL/6J mice triggered dose-dependent metabolic dysfunction in erythrocyte energy and redox homeostasis, accompanied by splenic metabolomic reprogramming and evident splenic extravascular hemolysis, suggesting radiation-induced peripheral blood cell destruction and secondary splenic hematopoietic compensation disorder (21). Non-human primate models further verified persistent hematopoietic genomic instability. Rhesus monkeys exposed to 1 Gy whole-body gamma radiation or C heavy-ion cranial radiation exhibited a seven-fold increase in lymphocyte chromosomal aberrations 24 h post-radiation, with sustained DNA damage detectable over long-term observational periods, providing evidence for the persistent genotoxic effects of space-type high-LET radiation on primate hematopoietic immune cells (29). Whole-body heavy-ion or X-ray radiation also induced dose-dependent splenic chromosome aberrations in mice, further supporting cumulative radiation-induced hematopoietic genomic damage (29).

Although current human spaceflight studies are limited to post-flight retrospective analyses without in-orbit dynamic monitoring, multi-evidential data from astronaut cohorts and ground-based simulation models collectively suggest that space radiation is a significant risk factor for persistent hematopoietic and immune system deterioration.

### 2.4 Systemic immune dysfunction secondary to bone marrow damage

Primary bone marrow hematopoietic suppression inevitably triggers systemic multi-level immune dysfunction, involving thymic developmental impairment, peripheral lymphocyte depletion, splenic immune dysregulation, and increased inflammatory susceptibility. As the primary lymphoid organ for T-lymphocyte maturation, the thymus is highly vulnerable to space radiation stress. Long-duration spaceflight significantly elevated endogenous urinary and plasma glucocorticoid levels in astronauts, accompanied by suppressed thymopoiesis and impaired migration of early thymic progenitor cells, resulting in persistent reductions in mature peripheral T-cell output and disrupted adaptive immune homeostasis.

Peripheral immune cell depletion represents another hallmark of radiation-induced immune injury. Human lymphocytes rapidly undergo interphase apoptosis within hours after radiation exposure exceeding 50 cGy, leading to acute peripheral lymphopenia (30, 31). Ground simulation studies further observed that combined space microgravity and radiation stress induces sustained splenic immune transcriptional dysregulation, with inflammatory and immune disorder-related gene profiles persistently altered for at least 1 week after stress cessation, alongside disrupted splenic B-cell differentiation programs analogous to human hematological disease signatures (32). Persistent splenic chromosomal instability and immune cell genome damage further exacerbate long-term immune dysfunction. Collectively, space radiation-induced primary bone marrow niche failure and HSC damage cause cascading impairments in thymic T-cell development, peripheral immune cell repertoire maintenance, and splenic immune regulatory function. This multi-organ immune suppression significantly elevates systemic inflammation and infection risk, constituting a critical health threat for long-duration deep-space exploration.

## 3 Impact of space radiation on the neural system

Long-term exposure to space radiation may harm the neural system, representing a recognized health concern with ongoing uncertainty regarding dose-response and severity, and remains a high-priority area for risk characterization (7, 33). The NASA Twins Study involved the administration of a Cognition battery, comprising 10 tasks, to the twins both before and after the flight. After data correction, results showed that astronauts' cognitive efficiency post-spaceflight was significantly lower than levels observed before and during the flight, with cognitive performance deterioration lasting for 6 months post-flight (26). Beyond the NASA twin study, exposing humans to high-energy charged particle radiation for research purposes is considered unethical. As a result, further investigation into the impacts of space radiation on the neural system is conducted utilizing rodents or neuronal cells (34). In this section, we deliberately restrict our analysis to mitochondrial alterations within the neural system. This focus reflects that mitochondria are both the earliest and most vulnerable targets of space radiation, directly linking acute oxidative injury to long-term neuropathology. Rodents or cells undergo radiation with either single or mixed high-energy particles to simulate exposure to space radiation. Preclinical studies in animal models have indicated that exposure to space-relevant radiation is associated with adverse neural effects, including cognitive and behavioral impairments and neurodegenerative-like changes, though human translation remains to be established (35–38).

**Figure 3:**
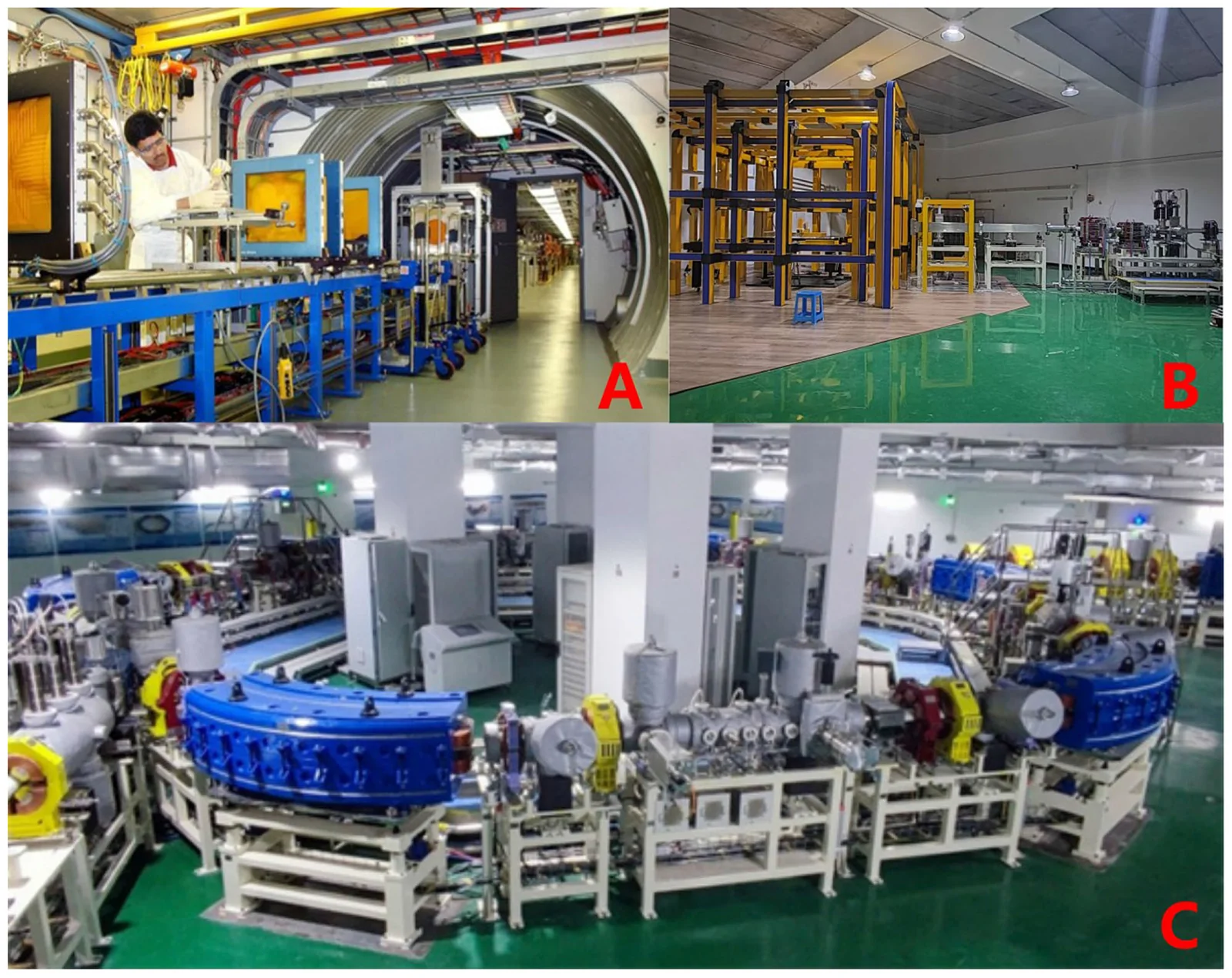
**(A–C)** Apparatus for simulating space radiation. **(A)** Sourced from NASA's GCRsim. **(B)** Originates from the terminal of the proton and heavy ion accelerator facility at Harbin Institute of Technology. **(C)** The accelerator module of the proton and heavy ion acceleration facility.

The Galactic Cosmic Ray simulator (GCRsim) was developed at Brookhaven National Laboratory's NASA Space Radiation Laboratory, with the aim of achieving comparatively realistic simulation of deep-space radiation conditions (39). GCRsim represents a simulated space radiation scenario that employs 33 high-energy ion beams, including ^1^H, ^4^He, ^12^C, ^16^O, ^28^Si, ^48^Ti, and ^56^Fe. The Space Environment Simulation Research Infrastructure in China, established by the Harbin Institute of Technology, is equipped to provide ion beams including ^1^H, ^4^He, ^12^C, ^18^Ar, ^36^Kr, ^73^Ta, and ^83^Bi. Recently, a growing number of researchers have employed analogous or simplified space radiation systems to investigate space life science (Figure 3).

Astronauts are confronted with potential neural hazards throughout prolonged orbital stays and upcoming crewed lunar and Mars expeditions (26, 40). Cumulative in-flight monitoring data and predictive risk modeling, together with rodent ground radiation experiments summarized in Table 2, collectively suggest plausible neural functional perturbations linked to deep-space radiation exposure, though inter-study variability persists across experimental platforms. High-LET space radiation particles are capable of penetrating nuclear chromatin and generating a spectrum of secondary protons, neutrons and γ-rays within target cells, with biological outcomes modulated by particle type, total dose and dose rate (41, 42). Such particle traversal frequently correlates with elevated cellular oxidative burden, which may drive the accumulation of ROS and reactive nitrogen species (RNS)—particularly within mitochondria, the primary organelle governing intracellular energy turnover—though the magnitude of this oxidative shift varies substantially by cell subtype and radiation regimen (43–45).

**Table 2:** Summary of representative findings from the last 5 years regarding the impact of space radiation on the neural system^a^.

Animal model (sex)	Age	Particle and dose	Dose rate	LET (Converted)	Assessment time point	Behavioral testing	Main conclusions	References
C57BL/6J mice (M)	6 months	Simplified GCR With 5 or 6 beams (5 or 30 cGy)	5 cGy/min	NR	6 weeks following radiation	NOR, EPM, FST, and MWM	GCR dose preferentially disrupts hippocampal inhibitory neurotransmission, perturbing oscillatory local field potentials and impairing learning, memory, and anxiety-related behaviors.	(107)
C57BL/6J mice (F)	6 months	^16^O (0.1 or 0.25 Gy)	0.18–0.33 Gy/min	~ 17 keV/μm	3 months after exposure	Y-Maze and NOR	Heavy-ion ^16^O radiation impairs object recognition memory and reduces hippocampal dendritic complexity in female mice.	(108)
C57BL/6J mice (M and F)	6 months	Simplified GCR with 5 beams (0.5 Gy)	3.1 Gy/min	~50 keV/μm	3 months following radiation	OF and NOR	GCR radiation induced cognitive deficits in mice, with amifostine more effectively restoring spatial habituation, object exploration, and novel object recognition in females.	(109)
C57BL/6J mice (M)	6 months	Neutron radiation (18 cGy)	0.5 Gy/h	NR	2 months after exposure	NOR, OiP, SIT, LDB, FST, and FE	Acute exposure to low-dose neutron radiation suppresses hippocampal synaptic signaling and impairs learning and memory in male mice.	(110)
C57BL/6J mice (F)	2 months	^56^Fe (20 cGy)	20 cGy/min	~151 keV/μm	3 months following radiation	EPM, MB, OF, SIT, and FST	Whole-body ^56^Fe radiation in adulthood enhances discrimination learning but impairs stimulus–response habit formation.	(111)
SHK mice (M)	2 months	^12^C (0.7 Gy)	1.6 Gy/min	~ 12 keV/μm	2 months after exposure	OF, Barnes maze, and NOR	^12^C expose induced heightened anxiety, impaired hippocampal-dependent memory retention, and reduced hippocampal cell numbers in mice.	(112)
C57BL/6J mice (M)	9–10 weeks	30-day spaceflight (N/A)	NR	NR	before and after the flight	Aerial drop test, OF, Rotarod, Grip force	Exposed 30-day spaceflight with elevated radiation, mice showed vestibular/motor deficits, heightened anxiety, and impaired learning.	(113)
Wistar rat (M)	6 months	^28^Si (5 cGy)	2–5 cGy/min	~50 keV/μm	12 ± 2 weeks post- radiation	String-pulling task	Single low-dose space radiation exposure impairs skilled hand movements and posture, indicating neural dysfunction.	(114)
Wistar rat (M)	NR	GCRsim or1 4He (10 cGy)	5 cGy/min	NR	72 h post- radiation	String-pulling task	Sensorimotor function and motivation/attention were impaired 72 h post ^4^He radiation.	(115)
C57BL/6J mice (M)	6 months	Measure GCR with 33 beams (75 cGy)	35-75 cGy/min	NR	4 months after exposure	EPM, OF, 3-CSI, and NOR	Irradiated mice displayed impaired sociability, diminished preference for social novelty, and attenuated social novelty-seeking behavior.	(116)
C57BL/6J mice (M and F)	6 months	Measure GCR with 33 beams (Total 50 cGy or 1 day 40 cGy)	2.08 cGy/d	NR	5 days after exposure	Dark and light open field session	GCR exposure induces significant and persistent deficits in CNS function in mice, with acute exposure causing more severe neurobehavioral impairments.	(117)
Wistar rat (M)	NR	γ-rays (9 Gy)	0.5 Gy/min	~0.9keV/μm	1 h, 1, 3, 7 & 10 days after exposure	None	γ-radiation induces oxidative stress, neuroinflammation, apoptosis, amyloid plaque deposition, neurotransmitter dysregulation, and reduced brain electrical activity.	(54)
Balb/c mice (M)	6 months	Simplified GCR with 5 beams (50 cGy)	NR	NR	3 months following radiation	Y maze and MWM	GCR radiation impaired short-term memory and quadrupled hippocampal chromosomal abnormality frequency compared to sham controls.	(49)
Wistar rat (M)	3 months	^12^C (0.14 Gy)	140 ± 15 mGy/min	10.3 keV/μm	25 days following radiation	LDB and MWM	Despite no detectable behavioral changes, radiation suppressed dopamine turnover, and lowered norepinephrine levels.	(118)
C57BL/6J mice (M and F)	23–24 weeks	Simplified GCR with 5 beams (5, 15, and 50cGy)	5 (0.4–0.9 cGy/min), 15 (0.9–1.2 cGy/min), or 50 cGy (1–1.3 cGy/min)	NR	72 h postradiation exposure	Composite Garcia NHBA	At 15 cGy, males and females showed sex-specific acute responses in species-typical behaviors; at 50 cGy, females exhibited delayed grooming.	(119)
C57BL/6J mice (M and F)	6 months	GCR with 33 beams (Total 50 cGy or 2 h 40 cGy)	2.08 cGy/d	NR	3 months following radiation	Object in updated location, object location memory, NOR, LDB, and SIT	Radiation exposure in space interferes with essential cognitive functions and the plasticity of neural networks.	(120)
C57BL/6J mice (M and F)	NR	Simplified GCR with 5 beams (0.75 Gy) and γ-rays (2 Gy)	112 mGy/min	0.8 keV/μm	3 days after exposure	OF, Rotarod, NOR, Spatial Novelty, and Y-Maze	GCRsim appeared to impair memory function in male mice and reduce total Aβ levels in both males and females.	(121)
C57BL/6J mice (F)	1 years	GCR with 33 beams (75 cGy)	50 cGy/min	NR	10 weeks after exposure	EPM, Gross locomotor activity, 3-CSI, OF, NOR, MB, and Nestlet shredding	Radiation exposure impaired stimulus–response learning and reduced the number of Doublecortin immature neurons in the dentate gyrus.	(122)
SD rat (M)	12 weeks	^1^H (10 cGy)	9 cGy/min	0.732 keV/μm	3 months after exposure	ATSET performance	Proton radiation impaired processing speed and cognitive flexibility in rats.	(123)
Wistar rat (M)	5 months	γ-rays (0.4 Gy) and ^12^C (0.14 Gy)	γ-rays 0.017 Gy/h & 0.14 Gy/h	γ-rays ~0.9 keV/μm & ^12^C 10.3 keV/μm	1–2 days after exposure	OF and Grip strength test	Radiation-induced striatal damage, coupled with elevated choline and reduced syntaxin 1A, contributes to hyperlocomotion in rats.	(124)

M, Male; F, Female; NR, not reported.

NOR, Novel Object Recognition test; EPM, Elevated-Plus Maze; LDB, Dark–light box; MWM, Morris Water Maze test; FST, Forced Swim Test; SIT, Social Interaction Testing; MB, Marble Burying; 3-CSI, Three-Chamber Social Interaction; OF, Open Field; OiP, Object in Place; FE, Fear Extinction Testing; NHBA, Neuroscore and Homecage Behavioral Analysis; ATSET, Attention Set-shifting Task.

^a^Translational limitation: Ground-based simulated space radiation experimental directly reflect radiation-specific biological effects but have interspecies translational limitations.

**Figure 4:**
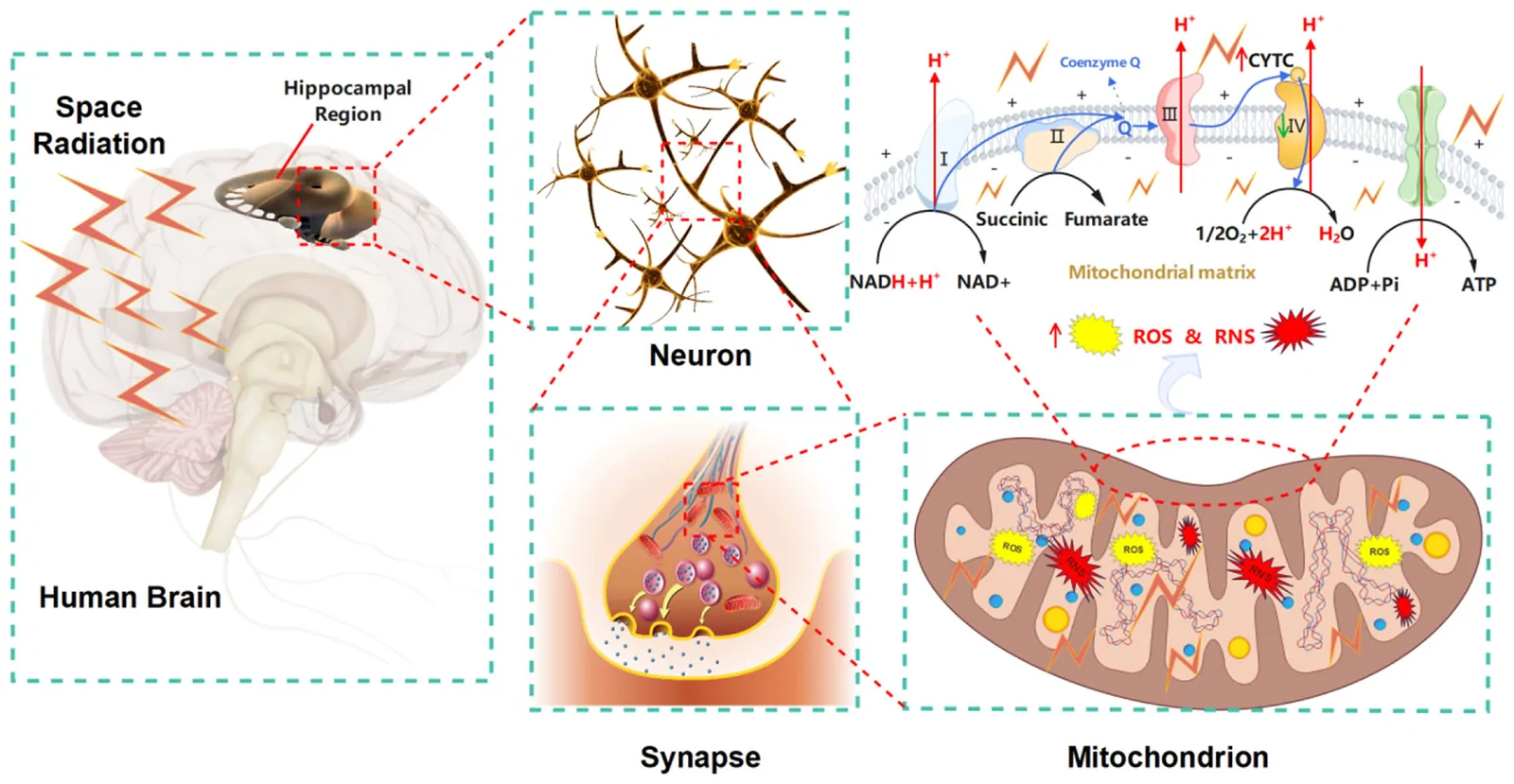
Mitochondrial dysfunction induced by space radiation impacts the neural.

Mitochondrial oxidative perturbation is hypothesized to be one major contributor to sustained neural functional alterations observed following space radiation exposure, yet the strength of this association differs across radiation quality and animal strain backgrounds (Figure 4) (17, 35, 46). Radiation exposure appears to extend electron residence duration within complexes I and III of the mitochondrial electron transport chain, which may raise the probability of off-target electron leakage to molecular oxygen; this leakage event could in turn facilitate the transcriptional upregulation of cytochrome c (Cyt c), a core respiratory chain mediator, with heterogeneous expression shifts reported across neural subpopulations (47, 48). Exposure to space radiation could extend the residence time of electrons in mitochondrial electron transfer chain complexes I and III, thereby increasing the probability of electron leakage to molecular oxygen. This process is associated with the aberrant upregulation of Cyt c expression, an essential element of the mitochondrial electron transport chain. The increase of Cyt c expression, as the primary electron carrier in the respiratory chain, directly disrupts mitochondrial energy metabolism homeostasis and redox balance, thereby triggering a long-term oxidative stress response (49). Oxidative imbalance and mitochondrial structural remodeling are often correlated with progressive cellular dysfunction, though the direction and magnitude of this coupling exhibit experimental heterogeneity. In murine tissue recovered from SpaceX-12 orbital flight, the inner mitochondrial membrane protein SURF1—a critical regulator of cytochrome c oxidase biogenesis—exhibited statistically significant downregulation, a molecular signature that may partially underpin neural mitochondrial impairment, while comparable expression shifts are not uniformly detected across all spaceflight rodent cohorts (50). Multiple radiation models indicate that space radiation-associated oxidative stress is often accompanied by mitochondrial structural damage and mtDNA lesions, which may remodel mitochondrial fission–fusion dynamics to variable degrees dependent on particle LET and delivered dose. For instance, whole-body 2 Gy 56Fe radiation in Kunming mice was associated with robust cerebral ROS accumulation; excess ROS may compromise neuronal antioxidant defense capacity, disrupt respiratory chain activity, and promote cytoplasmic Cyt c translocation, which can predispose cells toward apoptotic cascades, yet these responses show strain-specific divergence (51). Separately, low-dose ^1^6O or 56Fe particle regimens (10–50 cGy) have been associated with mild to moderate hippocampus-dependent memory deficits in select mouse lines, though cognitive impairment severity is inconsistent across sex and housing conditions. The mitochondrial biomarker translocator protein (TSPO), an outer mitochondrial membrane protein widely used as an *in vivo* imaging marker of neuroinflammation and reactive gliosis, tends to be upregulated in the hippocampus and prefrontal cortex following space-relevant heavy-ion radiation exposure (52, 53). This upregulation provides correlative evidence linking mitochondrial dysfunction to regional neuronal injury and neuroinflammatory activation, though it does not establish universal one-way causation (53). In rat models receiving 3 Gy whole-body γ-ray radiation, cortical and cerebellar tissue displayed variable degrees of oxidative dysregulation, attenuated neurotransmitter synthesis, and elevated TNF-α, Cyt c and caspase-3 concentrations. Radiation-triggered oxidative signaling may activate mitochondrial TNF-α receptors, which could accelerate Cyt c efflux and downstream caspase-3-mediated apoptotic execution, though the magnitude of this signaling axis varies by brain region and animal age (54).

Neural deficits induced by space radiation tend to manifest with prolonged, often persistent kinetics, in clear contrast to the transient sensory and motor declines triggered by microgravity, which commonly show partial functional recovery approximately 2 weeks after flight entry, with recovery efficiency differing by individual physiology. Notably, such sustained mitochondrial oxidative cascades and cytoplasmic cytochrome c translocation are not uniformly detectable across all deep-space radiation exposure regimes. Under extremely low chronic GCR dose-rate conditions, however, the likely reason for the failure of measurable mitochondrial ROS overproduction and robust Cyt c release to exceed baseline levels in rodents is the concerted upregulation of endogenous antioxidant defenses and mitochondrial quality control pathways (16). Furthermore, prominent sex-dependent divergent responses have been documented within identical low-dose mixed-field GCR paradigms: female rodents frequently exhibit partially suppressed mitochondrial oxidative cascades and blunted Cyt c mobilization relative to male counterparts, which may confer partial neuroprotective resilience against latent neural dysfunction (55, 56). As crewed lunar surface missions and long-duration Mars transit programs move forward, astronauts will face extended continuous radiation exposure windows, which may elevate cumulative neural injury risk to variable extents based on mission shielding design, total accumulated dose, particle LET composition, and individual biological traits including sex and baseline mitochondrial function. Accordingly, research frameworks ought to simultaneously evaluate both acute and latent neural health liabilities associated with deep-space radiation, while acknowledging inconsistent phenotypic readouts between high acute-dose ground radiation assays and low chronic-dose simulated GCR platforms, as well as sex-specific divergent molecular and behavioral endpoints observed across preclinical models.

## 4 Impact of space radiation on the gastrointestinal system

Space radiation negatively impacts the gastrointestinal system, particularly manifesting as intestinal dysfunction. The intestine is essential for sustaining homeostasis in the human body through its interactions with the immune, metabolic, and neurological systems. In the space environment, this organ serves as a primary target that significantly affects overall health. Current flight data indicate that changes in the gastrointestinal system during flight significantly affect the successful progression of astronauts' space missions. The gastrointestinal changes observed during spaceflight result from multiple interacting factors, including microgravity, radiation, and psychological stress. Due to the increasing risk of space radiation exposure in upcoming deep space exploration, it is imperative to systematically evaluate its impact on the gastrointestinal system. Space radiation can disrupt intestinal homeostasis, manifesting as dysbiosis, impaired barrier function, immune/inflammatory perturbations, and alterations in the gut-brain axis, primarily characterized in preclinical models. Moreover, intestinal dysfunction may lead to changes in intestinal metabolites, including short-chain fatty acids (SCFAs) (57–62).

Radiation first damages the physical structure of the intestinal mucosa. High-dose γ-ray (12 Gy) irradiation for 2 h rapidly induces apoptosis in mouse intestinal crypt cells, peaking at 12.5 h, which subsequently leads to excessive proliferation of regenerating crypt cells and the production of double-strand breaks and chromosomal instability (63). Under low-dose exposures more relevant to the space radiation environment, such as 0.5 Gy 56Fe particle irradiation, mouse intestinal epithelial cells exhibit persistent oxidative stress, elevated senescence markers, and reduced cell migration (64). Further studies show that high-LET radiation, including 56Fe and ^2^8Si, can induce senescence-associated secretory phenotypes in intestinal stem cells and upregulate inflammation-related signaling pathways such as p38, MAPK, and NF-κB (65, 66). At the molecular level, space radiation also downregulates base excision repair and nucleotide excision repair pathways, leading to sustained accumulation of DNA base damage (67). High-energy protons, the primary particle type in space, induce apoptosis in intestinal stem cells located in the basal or highly proliferative region, demonstrating a “supralinear” correlation with radiation dose. Ingenuity Pathways Analysis indicates that high-dose proton radiation can suppress XIAP- and TRAF1-mediated extrinsic and intrinsic apoptosis pathways (68). Apoptosis of intestinal stem cells is associated with a significant decrease in duodenal mucosal surface area in mice, which may result in acute gastrointestinal symptoms including nausea, vomiting, and diarrhea (68). The integrity of the intestinal barrier depends on the normal expression of tight junction proteins. In mouse samples exposed to the International Space Station, tight junction proteins were predominantly downregulated, compromising barrier function. Concurrently, genes linked to the IgA immune response pathway were significantly downregulated, and changes were noted in inflammatory bowel disease-related pathways, suggesting a potential risk of chronic inflammation. Metabolic function was also disrupted, as downregulation of the glucose transporter Glut2 and glycogen synthase Gys2 impaired normal intestinal metabolism. At the immune cell level, short-term spaceflight resulted in a decrease in MHC-II cell quantity within intestinal tissue, a reduction in lymphatic transport, and a compromised intestinal immune response (69–72). Intestinal stem cells may regulate the rapid self-renewal of intestinal epithelial cells, thus preserving the integrity of the intestinal barrier (61, 73). Radiation-induced stem cell apoptosis, inhibition of DNA repair pathways, oxidative stress, and senescence-associated phenotypes constitute the molecular basis of tissue damage. These histological and molecular injuries collectively form the pathological foundation of intestinal dysfunction.

The gut microbiome, residing in the human intestine (especially the colon), consists of approximately 1 × 10^1^4 microorganisms and is often termed the “second human genome.” It governs microbial metabolites—SCFAs—which serve as essential substrates for maintaining intestinal epithelium, modulating the immune system, and regulating inflammatory responses. The intestinal microbiome is susceptible to external environmental factors, suggesting it may be significantly impacted by high-energy particles (57, 74, 75). The NASA twin study revealed that spaceflight led to significantly altered gut bacterial composition at the genus level compared to ground controls, and at the phylum level, an increased Firmicutes/Bacteroidetes (F/B) ratio (26). Another study of nine astronauts residing on the International Space Station for 6–12 months found an elevation in *Parasutterella* and a decrease in *Fusicatenibacter, Pseudobutyvibrio*, and *Akkermansia*, accompanied by a slight increase in pro-inflammatory cytokines IL-8 and IL-1β, suggesting that spaceflight may induce a mild inflammatory immune response (76). In mice exposed to the International Space Station, the intestinal microbiome composition also changed, characterized by an increased F/B ratio, an elevation in *Clostridiales*, and a notable decrease in *Lactobacillales*, which are typically regarded as probiotics (69–72). Animal experiments further revealed differential effects of radiation dose and particle type on microbial community structure. After ^1^6O particle irradiation (0.1, 0.25, or 1.0 Gy), mouse intestines showed significant increases in *Akkermansia muciniphila, Erysipelotrichaceae*, and *Roseburia*, while *Ruminococcus gnavus* continued to decline, and multiple metabolic pathways (bile acids, arginine, methionine, and L-tryptophan) were disturbed (77). Proton radiation (0.5 or 1.0 Gy) led to increased microbial diversity, a reduction in beneficial bacteria such as *Akkermansia muciniphila, Faecalibaculum*, and *Lactobacillus*, and an increase in less common taxa like *Turicibacter* and *Clostridium*, along with significant decreases in intestinal methionine, S-adenosylmethionine, and glutathione levels, with notable alterations in the one-carbon metabolism pathway (78). At a higher proton dose (5 Gy), both α and β diversity decreased significantly in Balb/c and C57BL/6J mice, with 11 taxa showing notable differences (including *Muribaculaceae, Ruminococcaceae*, and *Lactobacillus*); metabolomics revealed 32 significantly elevated metabolites, implicating pathways such as the urea cycle, ammonia cycle, and α-linolenic and linoleic acid metabolism (79). Lethal-dose radiation induced sex-dependent dysbiosis, marked by an elevated F/B ratio and an enrichment of *Deferribacteres* (28). Inhibition of SCFA synthesis resulted in an increase in pro-inflammatory anaerobic bacteria and metabolites, persistent activation of AMP production, potential suppression of ATP production, elevated lipopolysaccharide-binding protein levels, and heightened risk of intestinal permeability. Males exhibited a decrease in *Bacteroidetes*, inhibition of SCFA synthesis, and an increase in lactic/pyruvic acid, leading to metabolic dysfunction. In contrast, females maintained functional homeostasis of the gut microbiome, which enhanced SCFA and tryptophan metabolism, thereby increasing radioresistance. The main mechanism contributing to male radiation sensitivity is a chronic deficiency of SCFAs (80, 81).

These alterations in the microbiome are not merely passive consequences of radiation exposure but carry profound biological implications that directly relate to astronaut health during long-duration deep-space missions. First, the reduction of beneficial bacteria (e.g., *Akkermansia muciniphila* and *Lactobacillus*) may weaken the protective function of the intestinal mucosal barrier and immune regulation, whereas an increase in opportunistic pathogens (e.g., *Clostridium*) could exacerbate local inflammatory responses. The elevated F/B ratio is generally associated with metabolic disorders and inflammatory states, and suppressed SCFA synthesis further compromises the energy supply and immune homeostasis of the intestinal epithelium, increasing intestinal permeability. This may predispose astronauts to bacterial translocation and systemic inflammation. Second, radiation-induced dysbiosis interferes with multiple key metabolic pathways (e.g., methionine, tryptophan, and bile acid metabolism). Alterations in these metabolites can affect the host's redox balance, immune responses, and neuroendocrine functions. For example, changes in tryptophan metabolites may influence mood and cognition via the gut-brain axis, while SCFA deficiency may impair epithelial repair and anti-inflammatory defenses. Third, the observed sex-dependent responses suggest that male and female astronauts may have different gut microbiome susceptibility to space radiation, necessitating individualized or sex-specific dietary or probiotic intervention strategies in future missions. Maintaining SCFA and tryptophan metabolic homeostasis could be a potential target for enhancing radiation tolerance. Finally, the interplay between microbiome changes and immune/inflammatory pathways may create a vicious cycle: dysbiosis promotes inflammation, inflammation further damages the intestinal barrier, and barrier disruption alters the microbial ecosystem, thereby exacerbating systemic health risks. Therefore, monitoring and modulating the gut microbiome during deep-space exploration is critical for safeguarding astronauts' gastrointestinal health, immune function, and even neurocognitive performance.

**Figure 5:**
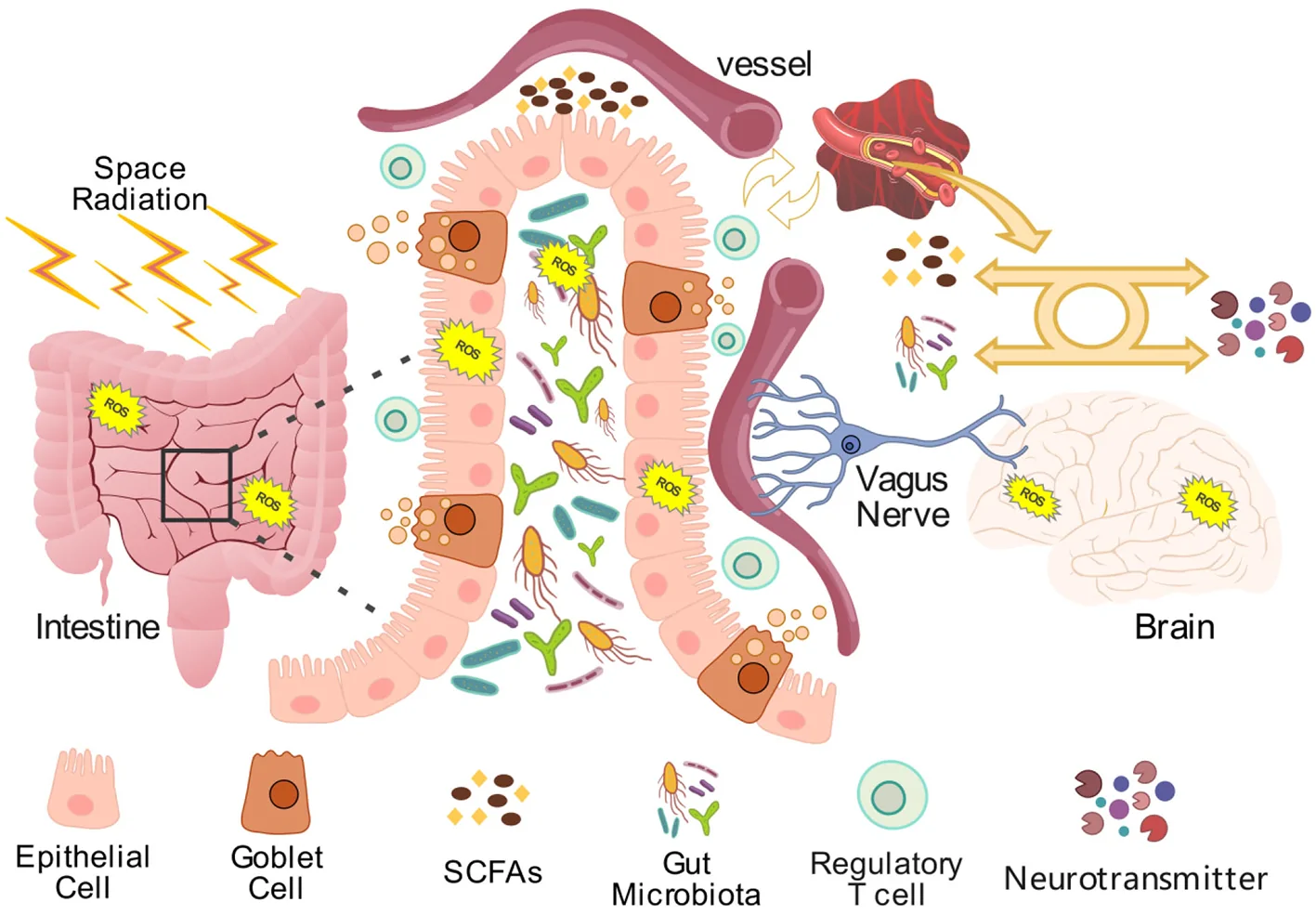
Interaction between the gastrointestinal and neural systems.

The gastrointestinal system serves as the locus for food digestion and absorption, as well as the largest immunological organ in the human body, regulated by the autonomic and enteric neural systems (Figure 5). The mucosa and lamina propria contain a high density of immune cells, including lymphocytes, plasma cells, macrophages, and dendritic cells, which constitute the primary line of mucosal immune defense. The gastrointestinal system includes an enteric nervous system, comprising enteric neurons and glial cells, which can function autonomously from the central nervous system (82–84). The impact of space radiation on the immune and neural systems related to the gastrointestinal tract is manifested, either directly or indirectly, through intestinal microorganisms or their metabolites, showing slight variations from prior discussions. Particular gut bacteria and their metabolites could affect the intestinal immune response governed by T regulatory cells (Tregs). The regulation of Tregs in the gut is mediated by *Helicobacter* species and GPCR43-related short-chain fatty acids (85, 86). The intestinal mucosa and lamina propria contain various innate immune cells, including macrophages and natural killer cells, which secrete cytokines that activate T cells, enhance adaptive immune responses, and initiate effective inflammatory responses to space radiation (87, 88). Ionizing radiation in radiotherapy can damage the intestinal mucosa and lamina propria, significantly disrupting intestinal microecology. This alters the microenvironment of Tregs, depletes factors including FOXP3, TGF-β, and CTLA-4, and markedly increases the absolute count of Tregs in the intestine. Consequently, this is associated with the conversion of Tregs into dysfunctional forms, which lose the ability to inhibit effector T cells (89, 90). Changes in the human gut microbiome have been identified as a significant contributor to several psychological and neurological disorders. The gut microbiome and brain function form a complex, dynamic bidirectional communication network, referred to as the gut-brain axis, which connects the neural and the gastrointestinal tract (91–93).

NASA conducted space radiation simulations using He particles at doses of 5 cGy and 25 cGy to systematically explore the gut-brain axis alterations in irradiated rats. Seven days after He radiation exposure, irradiated rats exhibited impaired social recognition memory accompanied by elevated plasma 5-hydroxytryptamine (5-HT) levels. Notably, approximately 90% of systemic 5-HT is peripherally synthesized in the intestinal tract, serving as a core signaling molecule of the gut-brain axis. Critically, circulating intestinal-derived peripheral 5-HT cannot penetrate the intact blood-brain barrier (BBB). Accordingly, elevated plasma 5-HT levels do not serve as direct evidence of enhanced central serotonergic neurotransmission, and the radiation-induced cognitive deficits observed in He-exposed rats cannot be attributed to direct peripheral 5-HT signaling within the brain (93, 94). Instead, peripheral intestinal 5-HT mediates gut-brain communication through two indirect BBB-independent regulatory pathways following space radiation exposure. The first is vagal afferent nerve transduction: enteric 5-HT activated by radiation stress binds to peripheral vagal nerve receptors, propagating electrical signals to the brainstem and further modulating neuronal activity in the hippocampus and cerebral cortex without the translocation of circulating hormonal molecules across the BBB (95–97). The second pathway relies on microbiome metabolite mediation: radiation-induced abnormal accumulation of intestinal 5-HT reshapes the metabolic profile of gut commensal bacteria, altering the production of BBB-permeable microbial metabolites including short-chain fatty acids (SCFAs) and tryptophan catabolites (67, 98). These metabolites readily cross the BBB to regulate cerebral neuroplasticity, modulate brain-derived neurotrophic factor (BDNF) expression, and trigger neuroinflammatory responses, ultimately contributing to cognitive dysfunction. Collectively, the elevated peripheral 5-HT and concomitant memory impairment in He-irradiated rats reflect disrupted vagal afferent signaling and dysregulated microbial metabolite-mediated gut-brain crosstalk, rather than direct central serotonergic activation (59). Low-dose radiation (LDR) also induces gut-brain axis dysfunction via microbial metabolic disturbance. A study in male Balb/c mice exposed to 0.5 Gy LDR revealed increased intestinal lipopolysaccharide (LPS) levels and depleted intestinal SCFA production, which were coupled with obvious cognitive impairment. Microbe-derived LPS and SCFAs serve as key mediators of LDR-induced neurocognitive deficits, and targeted regulation of these microbial metabolites effectively rescues cognitive dysfunction by reversing aberrant LDR-mediated Akt/mTOR signaling activation (99).

Gut-targeted radiation could affect protein expression in the brain through the microbiome-brain axis. Hatch et al. conducted a study on high-dose radiation exposure associated with acute radiation syndrome in the gastrointestinal tract of pigs, where male Sinclair minipigs were irradiated with 8 Gy of photons targeting the intestine. The findings showed that radiation led to a decrease in *Chlamydiae* and *Firmicutes*, while concurrently increasing the levels of *Coriobacteriaceae* and *Acinetobacter* in the mammalian intestine. PANX1 exhibited significant down-regulation in prefrontal cortex tissue, and modulation of the NLRP3 inflammasome pathway was observed. The alterations were closely linked to immune and neurotransmission functions (100). It should be noted that an 8 Gy photon radiation dose is mostly used to establish animal models of severe acute radiation sickness (101–103). Consistent with the radiation induced gut microbial and neurotransmitter dysregulation, Song et al. exposed mice to 4 Gy Co γ ray radiation and identified significant alterations in gut microbial composition and brain colonic functional homeostasis. Radiation exposure markedly reduced the relative abundance of Lactobacillus (involved in ACh and GABA metabolism) and increased the abundance of Streptococcus (modulating 5 HT and GABA). Stable intestinal microbiome homeostasis and balanced neurotransmitter metabolism are essential for cerebral neurotransmitter synthesis and the regulation of brain BDNF expression (68, 104). BDNF further modulates synaptic transmission to enhance presynaptic release of glutamate and GABA, thereby maintaining normal central neural function (105, 106). The dose level of 4 Gy or 8 Gy is roughly only applicable to research associated with extreme SPE and is generally unsuitable for scenarios involving routine deep-space exposure to GCRs.

The gastrointestinal system is highly sensitive, with minimal radiation exposure potentially leading to changes in the astronauts' intestinal microbiome, lesions in the mucosal layer and lamina propria, and impairment of the intestinal epithelial barrier function. The changes in astronauts' gastrointestinal systems during spaceflight are associated with alterations in immune and metabolic pathways that may, in turn, influence neurocognitive function via the gut-brain axis, although causal relationships remain to be fully established in humans. Intestinal health is a crucial defense mechanism against numerous diseases and disorders, especially during extended deep space exploration missions. Maintaining the homeostasis of the astronauts' gastrointestinal system is essential for their health security.

## 5 Discussion

Unlike recoverable low-LET radiation injuries, high-LET radiation (Including high-energy protons and HZE particles.) from GCRs causes long-term multi-organ damage and is a major unresolved health risk for deep-space exploration. Human datasets collected from LEO missions are confounded by concurrent flight stressors—microgravity, confined living conditions, circadian disruption, psychological strain, and dietary shifts—that complicate the isolation of radiation-specific phenotypes. By contrast, ground-based heavy ion and GCR simulation experiments under norm gravity serve as the benchmark for characterizing radiation-only injury signatures, while terrestrial low-LET radiotherapy cohorts exhibit poor translatability to deep-space radiation exposure owing to fundamental differences in particle species and dose delivery regimens. In fact, high-LET radiation produces potentially persistent pathological lesions which cannot be explained or mitigated by conventional low-LET radiation paradigms. Exposure to low-LET causes discrete DNA lesions that are readily repaired, while HZE particles cause dense clusters of DNA damage disrupting both DNA backbones and the chromatin structure in the vicinity and compromising the fidelity of both NHEJ and HR repair pathways. Genomic instability and long-lasting depletion of hematopoietic and thymic progenitor cells caused by faulty DNA repair led to persistent chromosomal aberrations and to long-term immune functional impairment in preclinical models. Moreover, high-LET radiation induces sustained mitochondrial dysfunction of immune cells and neurons, resulting in persistent electron leakage and chronic ROS/RNS overproduction (11–13). The chronic oxidative stress maintains chronic inflammatory signaling and cellular senescence for months after exposure. This potentially long-term damage cascade explains the latent neurocognitive and immune deficits in astronauts exposed to GCR.

Existing risk models underestimate the latent cumulative damage of high-LET radiation, and ignore the synergistic adverse effects of combined high-LET radiation and microgravity. In addition, the individual variability and sex-dependent radiation susceptibility have not been addressed by current astronaut protection frameworks, limiting the precision and individualized capacity of deep space health risk assessment (28, 81). In the future, high-fidelity low-dose-rate mixed-field GCR simulation models should be developed to reproduce authentic deep space radiation environments and revise current high LET risk assessment criteria and equivalent dose parameters (1, 46). Meanwhile, multi-omics analyses are needed to clarify individual and sex-specific mechanisms of high-LET radiation response to facilitate the development of individualized precision radiation protection. Moreover, human organ-on-chip platforms and physiologically relevant combined stress models that incorporate high-LET radiation and microgravity should be developed to bridge the translational gap between animal experiments and human spaceflight scenarios and to validate novel radioprotective strategies (18, 39). Obviously, high-LET radiation induces genotoxic, mitochondrial and immune damage that cannot be effectively addressed by conventional low-LET-oriented countermeasures, which is the core bottleneck for deep-space radiation safety. Further mechanistic exploration of high-LET-specific pathogenic pathways and development of specific, personalized protective approaches are essential to guarantee long-term astronaut health security in future deep-space missions.
